# Reproductive toxicity of manganese dioxide in forms of micro- and nanoparticles in male rats

**DOI:** 10.18502/ijrm.v17i5.4603

**Published:** 2019-06-26

**Authors:** Narges Yousefalizadegan, Zahra Mousavi, Tayebeh Rastegar, Yasaman Razavi, Parvaneh Najafizadeh

**Affiliations:** ^1^ Department of Pharmacology and Toxicology, Faculty of Pharmacy, Pharmaceutical Sciences Branch, Islamic Azad University, Tehran, Iran.; ^2^ Department of Anatomy, School of Medicine, Tehran University of Medical Sciences, Tehran, Iran.; ^3^ Cellular Molecular Research Center Iran University of Medical Sciences, Tehran, Iran.; ^4^ Department of Pharmacology, Faculty of Medicine, Iran University of Medical Sciences, Tehran, Iran.

**Keywords:** MnO2, Nanoparticle, Toxicity, Reproductive, Stereological study

## Abstract

**Background:**

Manganese Dioxide (MnO${}_{2}$) has long been used in industry, and its application has recently been increasing in the form of nanoparticle. **Objective:** The present study was an attempt to assess the effects of MnO${}_{2}$ nanoparticles on spermatogenesis in male rats.

**Materials and Methods:**

Micro- and nanoparticles of MnO${}_{2}$ were injected (100 mg/kg) subcutaneously to male Wistar rats (150 ± 20 gr) once a week for a period of 4 weeks, and the vehicle group received only normal saline (each group included 8 rats). The effect of these particles on the bodyweight, number of sperms, spermatogonia, spermatocytes, diameter of seminiferous tubes, testosterone, estrogen, follicle stimulating factor, and the motility of sperms were evaluated and then compared among the control and vehicle groups as the criteria for spermatogenesis.

**Results:**

The results showed that a chronic injection of MnO${}_{2}$ nanoparticles caused a significant decrease in the number of sperms, spermatogonia, spermatocytes, diameter of seminiferous tubes (p < 0.001) and in the motility of sperms. However, no significant difference was observed in the weight of prostate, epididymis, left testicle, estradiol (p = 0.8) and testosterone hormone (p = 0.2).

**Conclusion:**

It seems that the high oxidative power of both particles was the main reason for the disturbances in the function of the testis. It is also concluded that these particles may have a potential reproductive toxicity in adult male rats. Further studies are thus needed to determine its mechanism of action upon spermatogenesis.

## 1. Introduction

Human-made materials, especially those made with nanoparticles, have increased the public concern regarding their effects on the body health. Nanoparticles have very particular chemical, physical, and biological characteristics in their size, shape and a high proportion of surface to volume (1). Nano- and micro-metal oxides are recently used as MnO${}_{2}$ in different industries like Magnetic Resonance Imaging (MRI), drug delivery system, steel casting, catalysis, and consumer products such as batteries, which has undoubtedly led to the human exposure to these particles (2, 3).

Previous reports have shown that magnetic nanoparticles are biosafe and biocompatible and can be used in biomedical materials. Toxicological studies have also shown that these magnetic nanoparticles have adverse effects on the health of human beings as well as other living species. The biological safety of MnO${}_{2}$ nanoparticles is a controversial issue (4).

Previous studies indicated that MnO${}_{2}$ could increase the environmental risks and expose human beings to such disorders as Parkinsonism and occupational inhalation. The first report of chronic manganese poisoning was from a manganese ore-crushing plant in France (5). Moreover, invitro and invivo studies on MnO${}_{2}$ nano- and micro-particles indicated that they cause serious pathological risks to the central nervous system, respiratory system, cardiovascular and liver, as well as reproductive and developmental systems (6, 7).

Recent studies indicated that most nanoparticles insert harmful or toxic effects on spermatogenesis. It seems that such factors as the animal species, drug usage, nanoparticle's dose, and its characteristics (e.g., size, shape, chemical composition, surface, and surface charge) play an important role in determining the nanoparticles' effect on spermatogenesis (6). However, the penetration of nanoparticles to the blood-testis barrier is vital for explaining their toxicity in the process of spermatogenesis (7). Takeda observed sporadic seeds in the injured tubes of testis tissue and showed that Titanium Dioxide (TiO${}_{2}$) nanoparticles have a negative effect on spermatogenesis and epididymal sperm motility and induce histopathological changes appeared in the rat's testis (8). Studying the effect of gold nanoparticles on sperm and their penetration into the sperm cells revealed that these nanoparticles can result in the fragmentation of sperm's DNA. It was also shown that the presence of gold nanoparticles affected the sperms' motility; 25% of the sperms exposed to gold nanoparticles did not move (9).

Due to their critical role in the development and future well-being of wildlife, human exposure after maturity and also its health risks, reproductive and developmental toxicity have been used more in toxicology studies. Consequently, MnO${}_{2}$ nanoparticles are able to cross the blood-testis barrier and accumulate in the testis of rats which, in return, might have certain specific side effects.

Therefore, the aim of this study is to investigate the potential and possible toxic effects of MnO${}_{2}$ nano- and micro-particles on the reproductive system in male rats.

## 2. Materials and Methods

### Chemicals and dosage

All the chemicals and MnO${}_{2}$-MPs with size < 5 mm and purity ≥ 99% (CAS No. 1313-13-9) were purchased from Merck Company, Hohenbrunn, Germany. MnO${}_{2}$ nanoparticles, however, were synthetized by Dr. Safi Rahmani. The particles were scrutinized by an electron microscope to ensure that they were 25 to 85 nanometers in size. Both particles were dissolved in normal saline solution. The prepared solutions were sonicated for 30 min before injection. Nano and micro particle doses were selected according to previous investigations with pathological consequences on liver, kidneys, and testes of rats (10, 11).

### The MnO${}_{2}$ nanoparticles preparation

**Figure 1:**
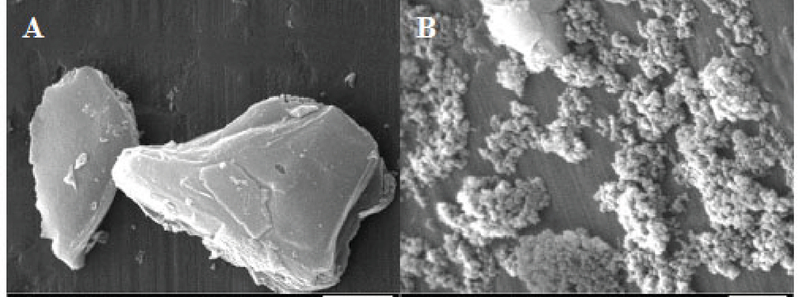
**A)** A scanning electron micrograph of MnO${}_{2}$ microparticles **B)** A scanning electron micrograph of MnO${}_{2}$ nanoparticles.

In this study, 20 ml of KMnO${}_{4}$ (0.2 mM/lit) were mixed with 16 ml MnO4 (0.125 mM/lit) for 5 min. The mixture was taken into a steel autoclave with Teflon covering and kept for 16 hr in 160°C. The brown product washed 3 times by distilled water and ethanol, and then dried (12 hr for 80°C). The size of synthetized particles were estimated 25 to 85 nanometers by an electron microscope (Figure 1). MnO${}_{2}$ nanoparticles were prepared using the hydrothermal procedure introduced by Zhang and his colleagues. (12)

### Animals and treatments

For the experimental study, 32 mature male albino Wistar rats weighing 180 ± 5 gr and age 8–10 wk were purchased from Shahid Beheshti University, Tehran, Iran. They were kept in an air-conditioned colony room with a light/dark cycle (temperature of 21–23°C and humidity of 30–40%) and were supplied with standard diet and enough water *ad* *libitum*. The rats were divided into four groups (n = 8) including control, vehicle (only received normal saline), nanoparticles (100 mg kg${}^{-1}$ per day), and microparticles (100 mg kg${}^{-1}$ per day). The treatment was carried out once a week for a period of 4 wk which was shown in previous studies to induce greater compassion in the testis.At the end of the treatment period, the rats were weighed and then anesthetized by ether. The reproductive organs including the left testicle, epididymis, seminal vesicle, and the left prostate were dissected and weighted after removing the lipid remnants. Then the blood samples were centrifuged (1500 rpm, 20 min) to measure estradiol, testosterone, follicle-stimulating hormone (FSH), and *luteinizing hormone (LH)* by immunoassay technique. (Orion Diagnostica; Finland and DRG Instruments GmbH; Germany).

### Assessment of the number of sperms

The sperm samples were obtained from the distal region (1.0 cm) of the left vas deferens and placed on a plate containing 2cc of Hank's Balanced Salt Solution and then were gently agitated at 37°C for 3 min. Specimens were spread on a hemocytometer, and the heads were counted manually under a light microscope. Data were expressed as the total number of sperm/ml:

Sperm count = No. of spermatozoa × Dilution factor × Depth factor No. of areas counted (13, 14).

### Sperm morphology

The sperm smears were stained by Eosin Y and evaluated in 10 microscopic fields, which the number of sperms 200-300 per animal. Spermatozoa were categorized as normal and abnormal. The abnormality was considered by a variety of head and tail abnormalities, containing blunt hook, banana-head, amorphous, pin-head, two-head, two-tail, small head, and bent tail. Finally, normal morphology sperm fraction parameter was defined as the mean number of normal sperm × 100/total number of sperm.

### Stereological study

The left testis was immersed in 10% formalin. Samples were divided into 5 micrometers and stained by hematoxylin and eosin method. The number of primary and secondary spermatocytes, spermatogonia, and diameter of seminiferous tubes were calculated per sample in 12 seminiferous tubes using a light microscope. The orientator method was used to obtain the Isotropic Uniform Random sections. Therefore, the testis was randomly placed on the φ clock which is divided into nine similar parts. A right cut was made along a randomly selected number (1-9). The first piece was then placed on the θ clock along with its previous cut surface on the 0-0 axis. The aforementioned procedure was repeated and the second piece was vertically placed on the θ clock so that its cut surface overlapped the 0-0 axis. A parallel cut was also made on this piece along with a accidentally selected number. Sections with 5 and 20 μm thickness were cut by the microtome after tissue processing and stained using Heidenhain's Azan method (15, 16).

### Ethical consideration

All experiments were performed in accordance with the guidelines for the care and use of laboratory animals (National Institutes of Health Publication No. 80-23, revised 1996) and were approved by the Research and Ethics Committee of the Pharmaceutical Sciences.

### Statistical analysis

The data were analyzed by ANOVA, Tukey Test, and Friedman Test with the statistical significance of p < 0.05 using the Stata software, version 13 (Stata Corp, College Station, TX, USA).

## 3. Results

### Effects of micro- and nanoparticles of MnO${}_{2}$ on clinical signs and body weight changes

**Figure 2:**
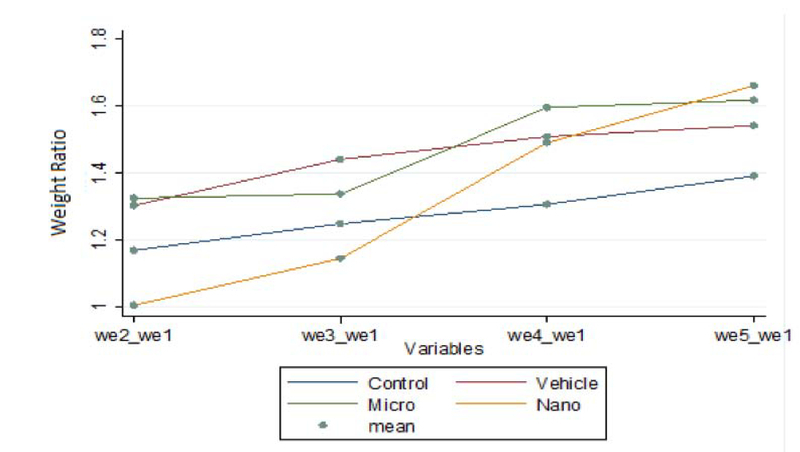
Comparison of changes in body weight per week relative to the weight of the first week, values expressed as mean for 8 rats.

All the animals survived the experiments. Figure 2 illustrates the body weight of rats in each group compared to their average body weight in the 1st wk of the experiment. The results of the Friedman Test indicated a significant difference between the experimental groups compared to the vehicle and control groups. The body weight gain of the animals treated with nanoparticles significantly decreased during the 2${}^{\mathrm{nd}}$ wk and increased in the 4${}^{\mathrm{th}}$ and 5${}^{\mathrm{th}}$ wk after injection, compared to the untreated control group.

The body weight gain of the animals treated with microparticles was continuous during the whole treatment and significantly increased compared to the untreated control group in the 4${}^{\mathrm{th}}$ and 5${}^{\mathrm{th}}$ wk after injection. The body weight gain of the rats receiving nanoparticles in most weeks was significantly lower than that of rats receiving the same dose of microparticles.

### The effect of micro- and nanoparticles of MnO${}_{2}$ on all genital organs 

The effect of micro- and nanoparticles of MnO${}_{2}$ on all genital organs compared to the body weight after 28 days of treatment in rats is shown in Table I. No significant difference was found in testis (p = 0.7), epididymis (p = 0.8), prostate (p = 0.6), and large and small diameter of left testis (p = 0.9) parameters among all groups.

### Effect of micro- and nanoparticles of MnO${}_{2}$ on epididymal sperm count and motility

The mean number of epididymal sperm showed a highly significant reduction in the nano- and micro groups compared to other groups (p < 0.001) (Table II).

The results of sperm motion analysis are shown in Table II. The percentage of immotile sperm significantly (p < 0.000) increased in the nano- and micro groups by 100% after 4 wk treatment compared to the vehicle and control groups.

**Table 1:** The effect of the MnO${}_{2}$ on the weights of prostate, seminal vesicle, left and right testicle, and diameter of left testis


**Groups**	**Left Testis (g)**	**Right Testis (g)**	**Left Epididymis (g)**	**Prostate (g)**	**Large diameter of Left Testis (mm)**	**Small diameter of Left Testis (mm)**
**Control**	0.69 ± 0.03	0.67 ± 0.03	0.024 ± 0.01	0.24 ± 0.01	19.9 ± 0.17	10.7 ± 0.05
**Vehicle**	0.61 ± 0.01	0.61 ± 0.003	0.23 ± 0.009	0.19 ± 0.03	20.14 ± 0.35	10.9 ± 0.08
**Micro-MnO${}_{2}$**	0.67 ± 0.09	0.57 ± 0.04	0.22 ± 0.02	0.22 ± 0.02	20.18 ± 0.39	11.3 ± 0.33
**Nano-MnO${}_{2}$**	0.60 ± 0.01	0.58 ± 0.01	0.22 ± 0.01	0.24 ± 0.03	20.12 ± 0.27	11.5 ± 0.17
Note: Values are means ± STD for 8 rats; MnO${}_{2}$: Manganese Dioxide						

**Table 2:** The effect of micro- and nanoparticles of MnO${}_{2}$ on sperm motility and epidydimal sperm count of rats


**Groups**	**Sperm Count (ml)**	**Sperm Motility (%)**			
	**Rapid**	**Slow**	**None**	**Immotile**
**Control**	316333 ± 27284	71	10	15	5
**Vehicle**	320666 ± 7218	61	18	11	10
**Micro-MnO${}_{2}$**	20666 ± 3051###	–	–	–	100${}^{****}$
**Nano-MnO${}_{2}$**	311266 ± 6241###	–	–	–	100${}^{****}$
Note: Values are means ± STD for 8 rats; ****p < 0.0001 compared to vehicle and control groups; ###p < 0.001 compared to the vehicle and control groups (one-way ANOVA followed by Tukey post-hoc test)					

### The effect of micro- and nanoparticles of MnO${}_{2}$ on the number of spermatogonia, spermatocyte, spermatid and seminiferous tubules

The mean number of spermatogonia showed a highly significant reduction in the micro- and nanoparticles of MnO${}_{2}$ groups compared to other groups (p < 0.001). Moreover, the reduction in the mean number of spermatocyte in the nano- and micro groups was highly significant when compared to other groups (p < 0.001). The mean of spermatid and seminiferous tubules diameter showed a highly significant (p < 0.001) reduction in the micro- and nanoparticles of MnO${}_{2}$ groups compared to other groups. A significant decrease (p < 0.05) was also observed in the mean spermatid and seminiferous tubules' diameter of nanoparticles of MnO${}_{2}$ group compared to the micro-group (Table III).

### The effect of micro- and nanoparticles of MnO${}_{2}$ on serum hormones level

Serum concentrations of FSH (p = 0.37), Estradiol (p = 0.8), and Testosterone (p = 0.2) in all four groups showed no significant difference (Table IV).

### Histopathological findings

**Figure 3:**
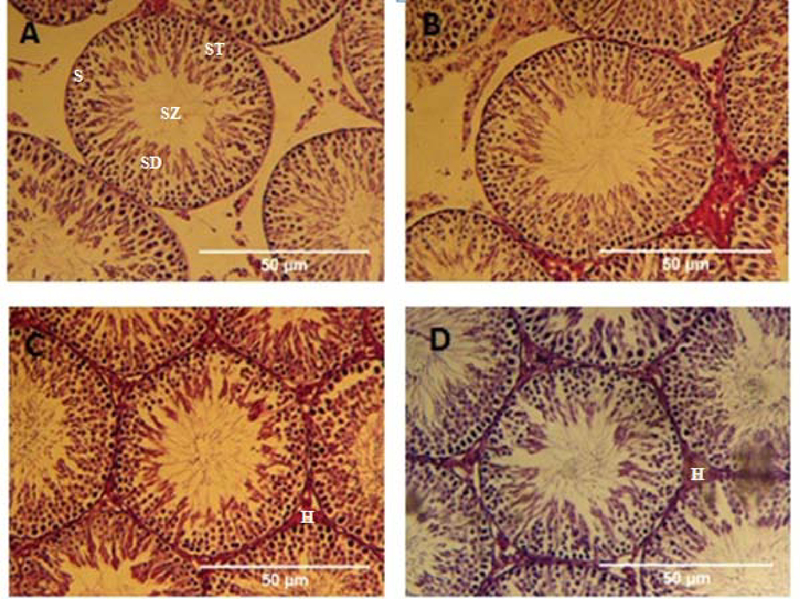
Histopathological micrographs of the testis's Control group (a), Vehicle group (b), Nanoparticles of MnO${}_{2}$ group (c), and Microparticles of MnO${}_{2}$ group (d). Germinal epithelium is normal in control and vehicle groups containing different germline cells. Spermatogonia(S), Spermatocyte (ST), Spermatid (SD), and Spermatozoa (SZ) in nano- and micro-MnO${}_{2}$ groups, the germline cells were also seen but the interstitial space hyalinized(H) because of fluid accumulation. All samples were stained with H&E (A, B, C, and D: ×10).

Histopathological micrographs of the testis show the structure and cellular arrangement of the seminiferous tubules. Germinal epithelium is normal in control and vehicle groups containing different germline cells, spermatogonia, spermatocyte, spermatid and spermatozoa. In nano- and micro-MnO${}_{2}$ groups, the germline cells were also seen but the interstitial space hyalinized because of fluid accumulation (Figure 3).

**Table 3:** Comparing the number of spermatogonia, spermatocyte, spermatid and seminiferous tubules in different groups of rats after 28 days of treatment


**Groups**	**Spermatogonia × 10${}^{6}$**	**Spermatocyte × 10${}^{6}$**	**seminiferous tubules × 10${}^{6}$**
**Control**	64.9 ± 0.26	203.9 ± 1.1	65.2 ± 0.86
**Vehicle**	64.96 ± 0.48	203.2 ± 0.28	64.7 ± 0.98
**Micro-MnO${}_{2}$**	50.6 ± 0.52${}^{***}$	162.1 ± 0.62	58.7 ± 0.31
**Nano-MnO${}_{2}$**	56.4 ± 0.31${}^{***\&\&\&}$	171.9 ± 0.50${}^{***\&\&\&}$	56.1 ± 0.6
Note: Values are mean ± STD; ***p < 0.001 compared to the vehicle and control groups; ${}^{\&\&\&}$p < 0.001 compared to the Nano-group; MnO${}_{2}$: Manganese Dioxide			

**Table 4:** The effect of micro- and nanoparticles of MnO${}_{2}$ on serum concentrations of FSH, estradiol, and testosterone


**Groups**	**FSH (µIU/L)**	**Estradiol (Pg/mL)**	**Testosterone(ng/mL)**
**Control**	1.75 ± 0.43	46 ± 2.8	0.85 ± 0.05
**vehicle**	3.6 ± 1.05	40 ± 5.7	4.01 ± 2.4
**Micro-MnO${}_{2}$**	4.9 ± 1.55	39.6 ± 6.01 22	2.91 ± 0.73
**Nano-MnO${}_{2}$**	3.4 ± 0.4	39.4 ± 4.04	4.37 ± 0.88
Note: Values are mean ± STD; MnO${}_{2}$: Manganese Dioxide; FSH: Follicle-stimulating Hormone			

## 4. Discussion

This study was an effort to investigate the effects of sub-chronic injection of MnO${}_{2}$ nanoparticles on spermatogenesis of male rats compared to the injection of MnO${}_{2}$ microparticles. The effects of MnO${}_{2}$ nano- and micro-particles on the body weight of rats during 4 wk of injection illustrated that the vehicle and control groups had a normal weight gain, but the experimental groups had a significant weight loss during the 2nd wk. They also had a significant weight gain after 2 wk compared to the vehicle and control groups. The previous studies demonstrated that the application of MnO${}_{2}$ using this method can cause hyperglycemia and hyperlipidemia; therefore, the significant weight gain of the experimental groups compared to the vehicle and control groups can be attributed to this phenomenon (3). Generally, the body weight of rats is measured to show their general health after nanoparticle injection. The increase of oxidative stress and metabolic disturbance as a result of free radicals have been mentioned as the main reasons for the decrease of weight gain after nanoparticle injection (17).

In the present study, the toxicity of MnO${}_{2}$ nanoparticles was investigated through subcutaneous injection. It was shown that these nanoparticles might cause the appearance of certain clinical symptoms such as weight loss. In this regard, Wang studied the high toxicity of nano- and microparticles of zinc powder in rats and reported severe symptoms of nausea, vomiting, and diarrhea in the rats treated with zinc powder nanoparticles in the first days of treatment. These findings were in agreement with the previous reports regarding the excessive oral prescription of zinc salts. Since the nanoscale materials are easily accumulated in different environments, it is believed that nano-scale zinc powder can also be easily accumulated in the body of animals (18).

Therefore, since the power of injected nanoparticles was stronger in inducing the oxidative stress, the effect of nanoparticles on the body weight was more significant. Another reason for the existing difference in the results can be attributed to different methods used in each study, including the rate, procedure, and period of injection (10). The findings of the present study showed that nano- and microparticles of MnO${}_{2}$ decrease the fertility factors such as the number of sperms, sperm motility, spermatogonia, spermatocytes, and diameter of seminiferous tubes. Since there have been no studies on the effects of MnO${}_{2}$ nanoparticles on the spermatogenesis, the effects of these nanoparticles have been compared to the effects of other forms of nanoparticles. Statistical analysis review of the effect of Mn${}_{2}$O${}_{3}$ nanoparticles on the serum level of sex hormones indicated that these particles significantly increased the hormones 14 days after treatment compared to the control group. This increase can be attributed to the effect of MnO${}_{2}$ nanoparticles on Leydig cells. Mn${}_{2}$O${}_{3}$ nanoparticles can also have a lower mitochondrial activity, leading to severe tissue damage and an increase in their secretion. On one hand, MnO${}_{2}$ nanoparticles increased the number of reactive oxygen molecules such as super oxidase as well as the oxidation of such molecules as proteins that accordingly leads to apoptosis (19). Several studies showed that nanoparticles can increase the transcription of mRNA as the steroidogenic acute regulatory protein of testis which influences the survival of Leydig cells and the production of steroid hormones (20). Moreover, the harmful effects of nanoparticles with their anti-androgenic features have been recognized as the cause of disturbances in the performance of the male reproductive system (testis tissue damage and sperm reduction). The steroidogenic acute regulatory protein plays an important role in regulating the cholesterol transfer into the inner membrane of mitochondria and increasing the steroid hormones (21, 22). On the other hand, it is assumed that silver nanoparticles can affect spermatogenesis and the number of spermatogenic cells and also the acrosome reaction in sperm cells; Miresmaeili. determined that nanoparticles have a negative effect on the spermatogenesis process and can influence reproductive potential in animal models (23). In the present study, the data indicated that FSH hormone level in those groups treated with nanoparticles significantly increased compared to the control group. The increase of FSH hormone level cannot be related to Gonadotropin-releasing hormone (GnRH). However, it can be attributed to the release of Inhibin hormone from Sertoil cells. Given the results of FSH hormone level, it is assumed that the feedback mechanisms are not applied just by the testis' steroids. But they play a role in regulating the FSH level by affecting the production of GnRH. The histopathologic study of testicular tissue sections with hematoxylin-eosin coloring in mice treated with zinc oxide nanoparticles with different doses of 1 and 2.5 ppm indicated various changes including, vacuolation of seminiferous tubes, reduction of germ cell lines in different stages of spermatogonia, irregularity in the order of germ cells, destruction of some seminiferous tubes involved in spermatogonia process, and the existence of colloidal liquid in lumen of seminiferous tubes. Lan and Ema showed that titanium dioxide nanoparticles could pass testicular blood barrier and accumulate in the form of granules in Sertoli cells (6, 24). This can accordingly lead to the reduction of Sertoli cells and thus damage and disorder of seminiferous tubes. Morphological changes of testis in animals treated with zinc oxide nanoparticles were observed after their exposure to nanoparticles. Spermatogonia process is disturbed due to the destruction of seminiferous tubes which is proved by the reduction of understudy parameters in this study (25).

Ema showed that TiO${}_{2}$ nanoparticles decrease the daily production and motility of sperms by causing disturbance in Sertoli cells (24). A study conducted in 2012 demonstrated that the treatment of mice with gold nanoparticles for 4 days had no adverse effects on testis epithelium and spermatogenesis (26). However, cytogenetic evaluations illustrated that gold nanoparticles could have affected the chromosomes of primary spermatocytes. Nevertheless, these nanoparticles caused no disturbance in chromosomes of spermatogonia cells. Another study, conducted in 2009, showed that gold nanoparticles had high toxicity power in spermatogenesis. These nanoparticles inhibit the motility of sperms. They also prevent the separation of chromatins and the formation of gametes (9, 27). In another study, the researchers demonstrated that the Fe${}_{2}$O${}_{3}$ nanoparticles destroy the seminiferous tubes and, in consequence, decrease the number of spermatogonia, primary spermatocytes, spermatids, and sperms (28). In 2013, a study on the evaluation of the toxicity of ZnO nanoparticles in spermatogenesis of mice showed that the spermatogenesis factors such as the number and motility of sperms changed significantly in the experimental group compared to the control group. ZnO nanoparticles decrease the diameter and height of the seminiferous tubes significantly and stop the maturity of sperm cell lines (7). Therefore, researchers concluded that ZnO nanoparticles can cause disturbance to spermatogenesis as a toxic compound. Kobayashi indicated a steady decrease in the number of alive and active sperms in relation to the increase of the amount of ROS (29). Therefore, the presence of ROS decreases the quality of sperms. This phenomenon occurs by the activation of oxidative stress by nanoparticles. According to this study and a other study, it can be mentioned that the chemical materials that are disruptive to endocrine system can cause oxidative damages to biomolecules such as DNA and protein through the production of free radicals (ROS) (26, 30). It is probable that free radicals cause mutation in testis, especially in spermatogonia, primary spermatocytes, spermatids, and spermatosoides, and can also seriously damage and destroy these cells (30).

## 5. Conclusion

The results of the present study demonstrated that nano- and microparticles of MnO${}_{2}$ decrease the number and motility of sperms significantly and reduce spermatogonia, spermatocytes, and the diameter of seminiferous tubes. Therefore, it seems that nano- and microparticles of MnO${}_{2}$ cause disturbance to spermatogenesis as toxic materials.

The present study showed that the prescription of MnO${}_{2}$ nanoparticles can cause significant changes in spermatogonia, sex hormones, and histology of male's testis that can also affect the performance of the male reproductive system and its fertility. Therefore, given the role of MnO${}_{2}$ nanoparticles in health product and medical equipment, they are considered as one of the harmful factors for reproductive systems that affect the fertility and, thus, should be evaluated and controlled with respect to their toxicity level in daily use.

##  Conflict of Interest

None of the authors have any potential conflict of interest of a funding source for this study.
